# Supplementary material for: Analysis of Il36a induction by C/EBPβ via a half-CRE•C/EBP element in murine macrophages in dependence of its CpG methylation level
Source: Genes Immun. 2021 Oct 25;22(7-8):313–21. doi: 10.1038/s41435-021-00153-5 (PMC8674125; doi:10.1038/s41435-021-00153-5)
Supplement: Supplementary file 1 — Supplementary Figure 1. Methylation sensitivity of C/EBPβ based on GSE98652 data. [file 41435_2021_153_MOESM1_ESM.pdf]

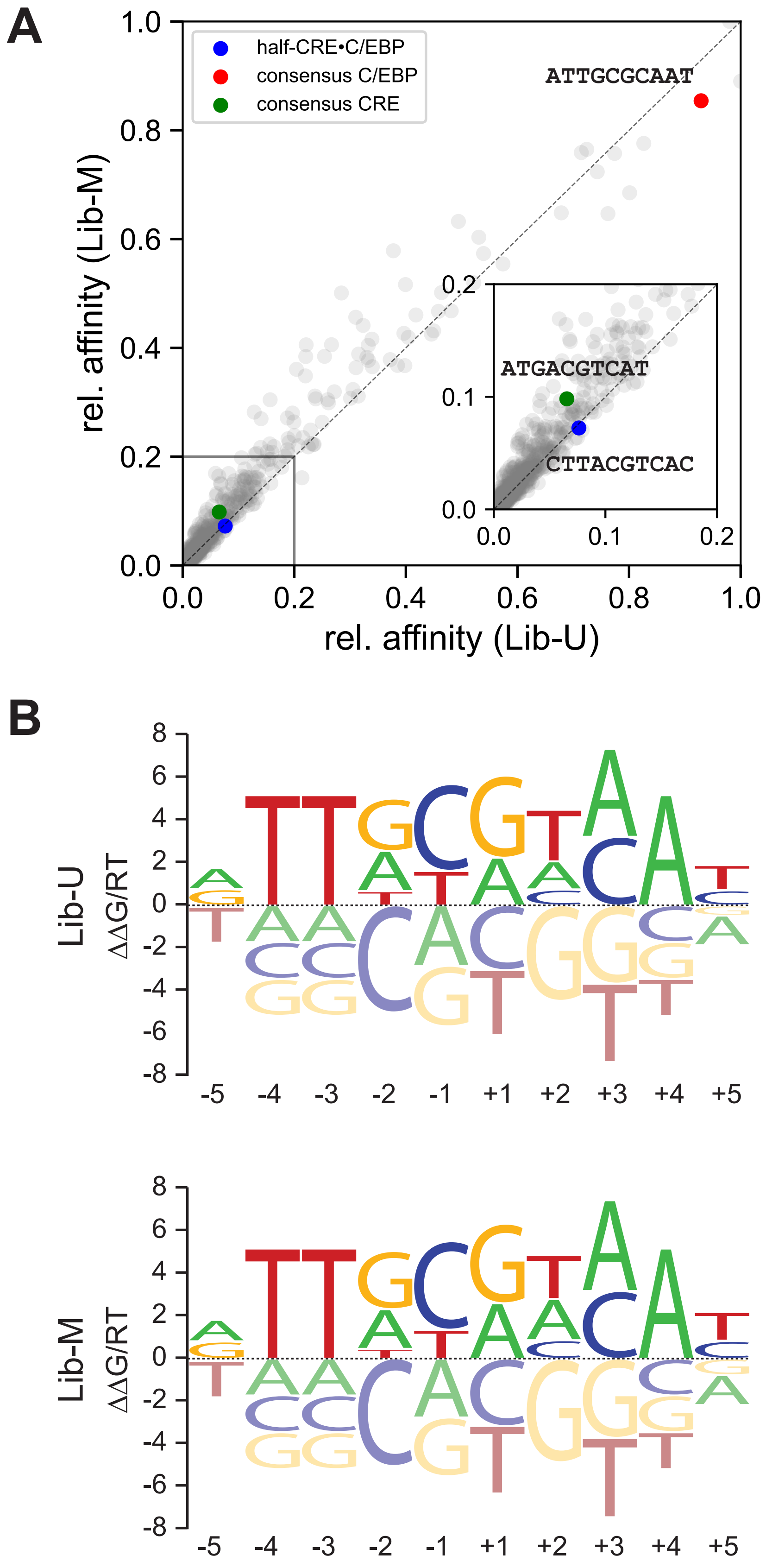

Supplementary Figure 1. Methylation sensitivity of C/EBPβ based on GSE98652 data. (A) Comparison of the relative enrichment of 10-bp oligonucleotides between the unmethylated library (Lib-U) and the methylated library (Lib-M) for C/EBPβ. The position of the half-CRE•C/EBP oligonucleotide is shown as blue dot, the position of the consensus C/EBP oligonucleotide is depicted in red and the position of the consensus CRE oligonucleotide is shown in green. **(B)** Energy logos for C/EBPβ derived from Lib-U (upper logo) and Lib-M (lower logo).
